# Characterizing the Use of Telepsychiatry for Patients with Opioid Use Disorder and Cooccurring Mental Health Disorders in Ontario, Canada

**DOI:** 10.1155/2018/7937610

**Published:** 2018-02-11

**Authors:** Brittanie LaBelle, Alexandra M. Franklyn, Vicky PKH Nguyen, Kathleen E. Anderson, Joseph K. Eibl, David C. Marsh

**Affiliations:** ^1^Northern Ontario School of Medicine, 935 Ramsey Lake Rd., Sudbury, ON, Canada P3E 2C6; ^2^Canadian Addiction Treatment Centers, 13291 Yonge St., Ste. 403., Richmond Hill, ON, Canada L4E 4L6

## Abstract

Rural patients with opioid use disorder (OUD) face a variety of barriers when accessing opioid agonist therapy (OAT) and psychiatric services, due to the limited supply of physicians and the vast geographic area. The telemedicine allows for contact between patients and their physician—regardless of physical distance.* Objective*. We characterize the usage of telemedicine to deliver psychiatric services to patients with OUD in Ontario, as well as traits of treatment-seeking patients with opioid dependence and concurrent psychiatric disorders.* Methodology*. A retrospective cohort study was conducted using an administrative database for patients who received psychiatric services via telemedicine between 2008 and 2014 and who also had OUD.* Results.* We identified 9,077 patients with concurrent opioid use and other mental health disorders who had received psychiatric services via telemedicine from 2008 to 2014; 7,109 (78.3%) patients lived in Southern Ontario and 1,968 (21.7%) in Northern Ontario. Telemedicine was used more frequently to provide mental health services to patients residing in Northern Ontario than Southern Ontario.* Conclusion*. Telemedicine is increasingly being utilized throughout Ontario for delivering mental health treatment. There is an opportunity to increase access to psychiatric services for patients with opioid dependence and concurrent psychiatric disorders through the use of the telemedicine.

## 1. Introduction

Nonmedical use of prescription opioids is a major public health crisis across North America [1]. From 2010 to 2011, 6% of the adult population reported nonmedical prescription opioid use, making opioids the second most prevalent nonprescribed drug following cannabis. This rate becomes further inflated among high-school students (15%–20%) and in marginalized populations [2, 3]. In Canada alone, opioid use disorder (OUD) and opioid-related overdoses account for 12% of deaths in patients between the ages of 25 and 34 [4]. Furthermore, in Ontario, opioid-related overdoses are responsible for higher death rates than all other nonprescribed drugs combined [1, 5].

Methadone and buprenorphine are long-acting synthetic opioid agonists prescribed to treat OUD [6]. Health Canada's Best Practice Guideline on Methadone Maintenance Treatment identifies opioid agonist therapy (OAT) with methadone or buprenorphine as key treatment and prevention strategies to manage OUD and its associated consequences [6]. OAT is associated with a reduction in the use of other substances, criminal activity, mortality, and risk behaviors for blood-borne pathogens [6]. Moreover, OAT leads to improvements in physical and mental health, social functioning, and quality of life among people with OUD [6].

In Ontario, patients who initiate OAT require supervised daily dosing of methadone or buprenorphine in a specialized addiction clinic, family physician's office, or pharmacy [7]. Patients residing in Northern Ontario face a variety of barriers when accessing these healthcare services, such as a lack of primary care physicians and the need to travel long distances to access care [7]. Considering the long-lasting nature of OAT, patients are required to stay connected with healthcare providers over an extended period of time and, consequently, patients receiving OAT are even more affected by the barriers of living in Northern Ontario.

The barriers experienced by patients seeking OAT mirror those experienced by patients seeking psychiatric services in Northern Ontario. As of 2009, the overall psychiatrist supply in Ontario was 15.7 psychiatrists per 100,000 people [8]. Delivery of health care in Ontario is provided across fourteen Local Health Integration Networks (LHINs). Southern Ontario is comprised of LHIN 1–12, and Northern Ontario is comprised of LHIN 13-14. Of the fourteen LHINs, Northern Ontario LHINs are far below average; North Western Ontario (LHIN 14) had the third lowest psychiatrist supply (approximately 7 per 100,000) and North Eastern Ontario (LHIN 13) had the sixth lowest psychiatrist supply (approximately 8.5 per 100,000) [8]. This problem is compounded by the vast geographic landscape of Northern Ontario, where many communities are isolated from larger urban centers. This—combined with the chronic shortage of physicians—leaves many Northern Ontario mental health patients without access to treatment.

To address these barriers, the provincial government invested in the Ontario Telemedicine Network (OTN), which is now one of the largest telemedicine networks in the world [9]. Telemedicine allows for the exchange of health information between providers and their patients from various locations across the province through two-way secure videoconferencing [9, 10]. The Ministry of Health and Long-Term Care reports that 49% of total telemedicine activity is used to service Northern Ontario [9]. According to the OTN, mental health and addiction medicine accounted for 72% of total patients served by telemedicine from 2012 to 2013 [11].

With a high prevalence of mental health disorders and many patients' needs being unmet, more research is required to better understand how access to mental health treatment can be improved, particularly for those patients seeking treatment in geographically isolated regions. In this study, we characterize patients with mental health disorders and concurrent OUD who receive psychiatric services via OTN. We also quantify resource usage by region, with a focus on Northern Ontario versus Southern Ontario.

## 2. Methods

### 2.1. Cohort Definition

We conducted a retrospective cohort study of all patients with OUD (as defined by having been engaged in OAT at some point during the study period) who also received psychiatric services for a mental health diagnosis other than substance use disorder via telemedicine from 2008 to 2014. Patients were at least 15 years or older and were residents of Ontario. Due to the nature of data collection (primarily derived from physician billing data), undiagnosed patients were not captured.

### 2.2. Data Sources

Data was accessed through the Institute for Clinical and Evaluative Sciences (ICES) through the Data Access Services division. The Ontario Drug Benefit (ODB) database was used to identify all patients engaged in OAT and to determine their past medication use of methadone or buprenorphine. The ODB database contains detailed records of all prescriptions dispensed to Ontario residents eligible for public drug coverage. In Ontario, residents are eligible for public drug coverage if they are aged 65 or older, reside in a long-term care facility, are disabled, are receiving social benefits for income support, or have high prescription drug costs relative to their net household income. Health system utilization was identified using the Canadian Institute for Health Information (CIHI) and the National Ambulatory Care Reporting System, and hospital admissions were identified using the CIHI Discharge Abstract Database. All diagnosis information from physician visits was determined using billing data from the physician Ontario Health Insurance Plan (OHIP) database. OHIP covers physician services for all permanent residents of Ontario. We obtained patient location of residence and demographic information from the Ontario Registered Persons Database, which contains a unique entry for each resident who has ever received insured health services. Patient information was linked anonymously across databases using encrypted 10-digit health card numbers. The linking protocol has been described extensively elsewhere [12, 13] and is used routinely for health system research in Ontario [14–16].

### 2.3. Telemedicine Definition

Patients' data were irrevocably stripped of personal identifiers before being made available for analysis. Telemedicine care via the OTN was identified by physician OHIP billing codes, which are specific to telemedicine appointments. Patients were included in the observational cohort if they had received a psychiatric diagnosis between 2008 and 2014 (listed in Table 1) from a psychiatrist via OTN (billing codes listed in Table 1) and if they were also diagnosed with OUD, as defined by having engaged in OAT at some point during the study period. It is worth noting that the treatment of OUD may or may not have involved physician care by OTN for the purposes of this study. Only patient data files with attachment to one of the 14 LHINs in Ontario were included in the analysis to aid subcohort analysis.

### 2.4. Methadone/Buprenorphine Subgroups

Patients with OUD were identified as any patient with a prescription for methadone or buprenorphine for the treatment of OUD during the study window. Patients receiving methadone or buprenorphine for whom the cost of medication was covered by the patient through direct payment or through private insurance or federal government insured health benefits were not identifiable in the data set. Previous research has demonstrated that the vast majority of patients treated with OAT in Ontario utilize the Ontario Drug Benefit program and therefore would be included in this analysis [17]. All patients were at least 15 years or older (to exclude data entry errors for newborns; patients < 18 years of age accounted for <1% of cohort) and were eligible for public drug coverage through the ODB plan. In Ontario, methadone is dispensed exclusively in liquid formulation, with very few exceptions; therefore, patients prescribed methadone in a tablet formulation (with a medication possession ratio greater than 20% over a one-year period) were excluded due to the likelihood that methadone was being administered for chronic pain management, despite being coded for addiction therapy in the billing records. We also excluded patients with missing information regarding place of residence, age, or gender. For evaluation of response to OAT, all patients were followed from their date of OAT initiation to the date of treatment discontinuation (patient did not receive a prescription for methadone or buprenorphine within 30 days of their last prescription), death, one-year follow-up, or end of the study period (December 31st, 2014).

### 2.5. Geographical Definition

Patient's postal codes were used to determine location of residence. Ontario is divided into 14 health care planning areas called Local Health Integration Networks (LHINs) for administrative, funding, and planning purposes. For geographic comparisons, patient data files with attachment to the North East (LHIN 13) or North West LHINs (14) were included in the Northern Ontario group for analysis compared to the remaining LHINs for Southern Ontario.

### 2.6. Costing

In order to calculate costs of OTN, the following variables were considered: inpatient hospitalization, same day surgery, National Ambulatory Care Reporting System (visits to ED, dialysis clinics, and cancer clinics), ODB, rehabilitation, complex and continuing care cost, home care services, OHIP physician billing, OHIP lab billings, OHIP nonphysician billings, OHIP shadowing billings, Family Health Organization/Family Health Network physician capitation cost, long-term care cost, Ontario Mental Health Reporting System Metadata admissions to designated mental health beds, and assisted device costs.

### 2.7. Analysis

Descriptive statistics were summarized for baseline characteristics of patients, and standardized differences were used to compare characteristics between Northern Ontario and Southern Ontario patients. Standardized differences < 0.1 are generally not considered to be meaningful [18]. All statistical analyses were carried out using SPSS (V.22). We used ArcGIS to produce maps illustrating the distribution of telemedicine delivery and distance between patients and their provider for each LHIN. Geographic boundaries by LHIN were retrieved from Statistics Canada [19].

### 2.8. Ethics Review

This study was approved by the Research Ethics Board of Laurentian University, Sudbury, Ontario, and by Sunnybrook Hospital, Toronto, Ontario.

## 3. Results

From 2008 to 2014, we identified 9,077 publicly insured patients who had received OAT and who received a mental health diagnosis from a psychiatrist through telemedicine. Of these, 7,109 (78.3%) lived in Southern Ontario and 1,968 (21.7%) lived in Northern Ontario. Patient characteristics by geographic location are summarized in Table 3. When comparing patients residing in Northern Ontario to those in Southern Ontario, it was found that Northern Ontario contains a concurrent disorder patient cohort with higher percentage of females (52.74% versus 45.17%), less education (23% high school completion rate versus 27%), greater impoverishment (47.46% in lowest income quintile versus 42.90%), and less enrolment in primary care (46.70% versus 51.68%).

**Figure 1:**
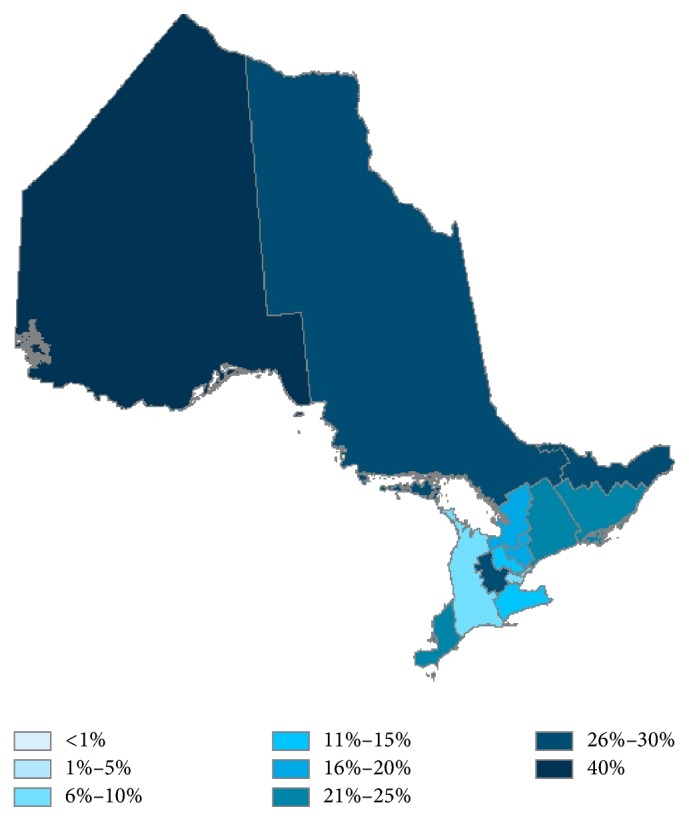
Percent of patients with concurrent disorders receiving psychiatric services via telemedicine.

Telemedicine is used more frequently to deliver psychiatric services in Northern Ontario than Southern Ontario for patients with concurrent OUD (Figure 1). In fact, telemedicine accounts for 26% to 40% of all psychiatry delivery to patients with concurrent disorders living in Northern Ontario LHINs; this number ranges from 1% to 30% in Southern Ontario LHINs (Figure 1). Overall, when moving from Southeastern Ontario towards Northwestern Ontario, there is increased telemedicine usage for delivering psychiatry to patients with concurrent disorders.

**Figure 2:**
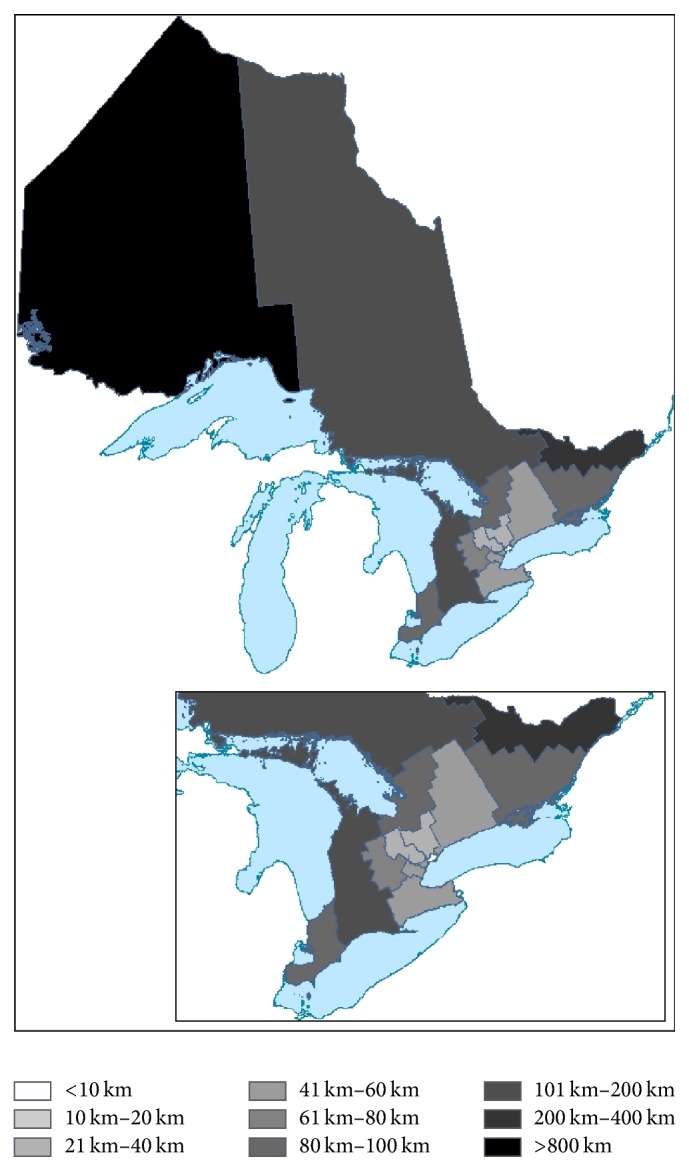
Median distances (km) between the residence of patients receiving psychiatry via telemedicine and their provider.

Concurrent disorder patients residing in Northern Ontario live farther away from their physicians (Figure 2) (median distance 341 km; interquartile range 73 km–901 km) compared with patients residing in Southern Ontario (median distance 75 km; interquartile range 33 km–142 km) (Table 1). Moreover, patients live a greater distance from their psychiatrist when they are receiving their services through telemedicine. In fact, LHIN 14 has the largest median distance between patients receiving mental health services and their provider (median distance 900 km), followed by LHIN 11 (median distance 322 km), LHIN 2 (median distance 136 km), and LHIN 13 (median distance 122 km) (Figure 2). LHIN 7, Toronto Central, has a median distance of only 4 km between patients and psychiatrists when receiving psychiatric services via telemedicine and it also has less than 1% of its patients serviced by telemedicine.

The number of concurrent disorder patients receiving psychiatry via telemedicine has increased from 347 to 5,879 over a period of six years (Table 2). The mean cost per patient of delivering psychiatric services to patients via telemedicine has also increased during this period; therefore, the total cost to the health care system of using telemedicine for mental health services has increased as well. However, we are not able to describe the costs avoided through the provision of care by telemedicine compared to transportation of patients to see the psychiatrist in person within the data utilized for this analysis.

## 4. Discussion

In the present study, we found that patients with concurrent disorders receive psychiatric services via OTN more often than patients who do not have OUD. Overall, telemedicine is currently heavily utilized for psychiatric service delivery, particularly in Northern and rural regions of Ontario. Notably, OTN is utilized more frequently in the geographically dispersed regions of Northern Ontario.

In Ontario, opioid agonist therapy can be delivered remotely via telemedicine where the physician is remote and a nurse, pharmacist, or clinic staff interact with the patient and dispense observed or carried methadone or buprenorphine doses. While there are no set determinants where virtual addiction medicine clinics can be established, historically, Northern, rural, and remote regions were almost exclusively serviced by telemedicine [7]. More recently, virtual clinics are also being established in urban centers due to the enhanced efficiency of allowing a single physician to service multiple clinic locations regardless of the clinic location or setting.

The heightened demand for telemedicine in isolated regions indicates a need to provide a model of care that overcomes geographical isolation. For this reason, telemedicine is particularly important in Northern Ontario, where there are geographic barriers to accessing services due to the uneven distribution of limited physician supply in a vast geographic area. Similar to patients enrolled in OAT [7], the concurrent disorder cohort residing in Northern Ontario live a greater median distance from their physicians than patients in Southern Ontario. To address this issue, telemedicine can provide a platform for physicians to provide psychiatric care to patients living further away with little to no travel for both the physician and patients.

We also found that the number of patients receiving psychiatry via telemedicine increased from 2008 to 2014. This can be explained by a number of factors, including the increased demand for psychiatry and the billing incentive [20] that is associated with providing treatment via telemedicine. The increase in OTN has resulted in better access to mental health services—including OAT—throughout Northern and Southern Ontario and a decrease in travel time for patients [10, 21]. In addition, the Ontario Ministry of Health and Long-Term Care have reported that Northern travel costs have been reduced by an estimated $25,000,000 annually [9].

Telemedicine is a key solution for increasing access to mental health services for patients with concurrent disorders, particularly for those patients living in isolated regions. A recent review found not only that telemedicine increases access to psychiatric services, but that it is effective from the perspective of the patient, provider, program, and society as a whole [22]. Patients across diverse clinical populations and receiving a wide-range of services have reported high levels of satisfaction with telemedicine [23]. For example, Lindsay et al. (2015) reported that psychotherapy delivered via telemedicine has similar results as traditional in-person treatment with regard to treatment outcomes, therapeutic relationship, and retention [24]. Moreover, mental health services provided via telemedicine are clinically superior to reduced or no mental health services [23]. A recent study on treatment outcomes in OAT found that patients receiving services via telemedicine had levels of substance use, time to abstinence, and treatment retention rates that were equal to patients receiving OAT face-to-face [25].

Altogether, as many as 55% of patients receiving OAT have a concurrent mental health disorder [26]. Importantly, without treatment that targets both OUD and other psychiatric symptoms, patients have higher rates of continued substance use and overdose, as well as decreased occupational functioning and quality of life [27–29]. Studies have demonstrated that telemedicine-delivered OAT for OUD is an effective treatment modality [20]; therefore, it is reasonable to conclude that telemedicine may also be an effective platform to coordinate mental health care and OAT for patients with concurrent mental health and OUD.

Our study has some limitations that should be noted. Using the data from ICES, we were able to obtain an accurate and complete picture of telemedicine usage for psychiatry delivery; however, because Ontario's publically funded drug benefit plan only covers patients aged 18–65 who are on social assistance, we did not capture patients whose methadone or buprenorphine medication cost was covered by the patient directly or through private insurance or federal government funded insured health benefits. Considering that this study only includes patients who are provincially insured, the lowest income quintiles may be inflated and, consequently, may not entirely represent the clinical population of patients with OUD and concurrent mental health diagnoses. We were also limited in capturing the true opioid dependent population, as only treatment-seeking patients would be captured in our definition. Patients were only included if they had received a prescription for methadone or buprenorphine within the timeframe; however, a patient with OUD who was not seeking treatment would not have met these criteria. Additionally, given the nature of secondary data, we were unable to evaluate the patient experience, including whether the patient was satisfied with OTN care that they received.

This study also has many strengths, one of which being its large sample size. With over 9,000 patients in our study, we were able to characterize the use of telemedicine to care for patients with OUD and concurrent mental health disorders, further contributing to the existing literature on telemedicine in Ontario. More specifically, we have focused our study on patients who are accessing psychiatry in Northern Ontario, an area that is not well studied. Given the psychiatrist shortage in this region of the province—and the common cooccurrence of OUD in this population—it is important to better understand how telemedicine is being utilized to provide psychiatric care to patients with concurrent disorders throughout the province. Patients in Northern Ontario are often faced with a variety of barriers when accessing treatment; understanding the utilization of telemedicine in this geographic area may enhance care for this patient population.

Telemedicine is increasingly being applied throughout Ontario for delivering mental health treatment to patients with concurrent disorders. Increasing the access to psychiatric care delivered via telemedicine may improve clinical outcomes for patients with OUD and concurrent psychiatric disorders. This is especially true of Northern and rural areas, where physician supply is limited. Future qualitative studies may aid in measuring telemedicine care outcomes, including patient's perspective of whether OTN achieves the same results as face-to-face care.

The application of telemedicine-delivered mental health and addiction services are very well suited in rural and remote settings. In Ontario, virtual clinics are often employed to service remote communities and include some clinics operating on First Nation reserves in collaboration with the First Nation leadership. While the present study is based on the Ontario context, we believe the findings are generalizable due to high quality video conferencing options being widely available with broadband Internet. It is important to note that not all remote communities will have access to broadband service, and in those instances alternative models may be more appropriate.

## Figures and Tables

**Table 1:** ICD-9 mental health classifications.

291: alcoholic psychosis, delirium tremens, Korsakov's psychosis
292: drug psychosis
295: schizophrenia
296: manic depressive psychosis, involutional melancholia
297: paranoid states
298: other psychoses
300: anxiety neurosis, hysteria, neurasthenia, obsessive compulsive neurosis, reactive depression
301: personality disorders (e.g., paranoid personality, schizoid personality, obsessive compulsive personality)
302: sexual deviations
303: alcoholism
304: drug dependence, drug addiction
305: tobacco abuse
307: habit spasms, tics, stuttering, tension headaches, anorexia nervosa, sleep disorders, enuresis
309: adjustment reaction
311: depressive or other nonpsychotic disorders, not elsewhere classified
313: behaviour disorders of childhood and adolescence
314: hyperkinetic syndrome of childhood

**Table 2:** Utilization of telemedicine for psychiatry and mean cost to the health care system.

Year	Total number of patient visits	Number of unique patients	Mean (± SD) cost to the system per patient
2008	1,588	347	6,455.64 ± 11,268.20
2009	4,768	943	7,255.15 ± 12,189.21
2010	11,507	1,748	7,852.72 ± 12,868.25
2011	22,998	3,175	8,417.49 ± 13,189.38
2012	46,150	4,300	9,003.45 ± 15,719.65
2013	56,261	5,254	9,605.53 ± 17,107.52
2014	58,863	5,879	-

**Table 3:** Characteristics of patients receiving psychiatry via telemedicine by geographic location.

Variable	Overall *N* = 9,077	Southern Ontario *N* = 7,109	Northern Ontario *N* = 1,968
Age, yr			
Median (IQR)	36 (29–45)	36 (29–45)	35 (28–44)
Gender, *N* (%)			
Female	4,249 (46.81%)	3,211 (45.17%)	1,038 (52.74%)
Male	4,828 (53.19%)	3,898 (54.83%)	930 (47.26%)
Median (IQR) distance between patient residence and physician address (km)	86 (36–262)	75 (33–142)	341 (73–901)
Median percentage of high school completion, % (IQR)	26 (21–31)	27 (22–32)	23 (19–27)
Number enrolled in primary care, *N* (%)	4,593 (50.60%)	3,674 (51.68%)	919 (46.70%)
Income quintile, *N* (%)			
Missing	23 (0.25%)	15 (0.21%)	8 (0.41%)
1	3,984 (43.89%)	3,050 (42.90%)	934 (47.46%)
2	2,058 (22.67%)	1,664 (23.41%)	394 (20.02%)
3	1,385 (15.26%)	1,089 (15.32%)	296 (15.04%)
4	981 (10.81%)	789 (11.10%)	192 (9.76%)
5	646 (7.12%)	502 (7.06%)	144 (7.32%)
